# Supplementary material for: Response Predictive Markers and Synergistic Agents for Drug Repositioning of Statins in Ovarian Cancer
Source: Pharmaceuticals (Basel). 2022 Jan 21;15(2):124. doi: 10.3390/ph15020124 (PMC8880614; doi:10.3390/ph15020124)
Supplement: Supplementary file 1 [file pharmaceuticals-15-00124-s001.zip › pharmaceuticals-1474774-supplementary/Table S1.pdf]

Table S1

Regulatory genes affected by simvastatin compared with those affected by L-778123 in OVSAHO and KURAMOCHI cells.

No	Gene Symbol	Fold change	
		OVSAHO_Simvastatin compared with OVSAHO_L778123	OVSAHO_Simvastatin compared with KURAMOCHI_Simvastatin
Top 10 up-regulated genes			
1	*PIK3IP1*	10.94	1.00
2	*BMF*	7.41	0.955
3	*MGP*	5.88	0.807
4	*ERVH-3*	5.82	1.08
5	*RARA-AS1*	5.79	1.04
6	*PRSS57*	4.68	0.819
7	*HMGCS2*	4.65	1.18
8	*SEMA6D*	3.86	1.04
9	*N4BP2L1*	3.67	0.919
10	*EMP3*	3.62	0.918
Top 10 down-regulated genes			
1	*GSTA9P*	0.0914	1.12
2	*MID1*	0.113	1.18
3	*ERVMER34-1*	0.120	0.903
4	*ZG16*	0.154	1.15
5	*RABGEF1*	0.158	1.06
6	*WDR62*	0.175	0.905
7	*E2F7*	0.179	1.05

8	*SGO1*	0.194	0.828
9	*HELLS*	0.203	1.06
10	*SNAR-G1*	0.203	1.16
