# Supplementary material for: Response Predictive Markers and Synergistic Agents for Drug Repositioning of Statins in Ovarian Cancer
Source: Pharmaceuticals (Basel). 2022 Jan 21;15(2):124. doi: 10.3390/ph15020124 (PMC8880614; doi:10.3390/ph15020124)
Supplement: Supplementary file 1 [file pharmaceuticals-15-00124-s001.zip › pharmaceuticals-1474774-supplementary/Table S2.pdf]

Table S2. Gene ontology (GO) processes regulated by simvastatin in OVSAHO and KURAMOCHI cells.

GO Terms	GO ID	Genes in category	Genes in list	p-value
**UP**				
Biological Process*				
cell cycle	7049	2748	113	2.76.E-27
cell cycle process	22402	2011	95	6.20.E-27
mitotic cell cycle process	1903047	1245	72	3.25.E-25
chromosome segregation	7059	536	47	7.39.E-24
mitotic cell cycle	278	1478	76	1.73.E-23
DNA replication	6260	426	39	1.40.E-20
chromosome organization	51276	1806	78	1.62.E-19
nuclear chromosome segregation	98813	465	39	3.08.E-19
sister chromatid segregation	819	364	34	2.49.E-18
cell cycle phase transition	44770	853	50	9.10.E-18
Cellular component*				
chromosome	5694	1485	78	7.61.E-25
chromosomal part	44427	1273	70	2.28.E-23
condensed chromosome	793	331	32	7.57.E-18
chromosomal region	98687	519	39	1.07.E-17
chromosome, centromeric region	775	298	29	2.35.E-16
non-membrane-bounded organelle	43228	6203	150	5.10.E-14
intracellular non-membrane-bounded organelle	43232	6203	150	5.10.E-14
condensed chromosome, centromeric region	779	175	21	5.60.E-14
kinetochore	776	202	22	1.05.E-13
condensed chromosome kinetochore	777	154	19	5.46.E-13
Molecular Function				
catalytic activity, acting on DNA	140097	270	20	1.34.E-09
actin binding	3779	666	31	4.28.E-09
cytoskeletal protein binding	8092	1395	48	5.03.E-09
DNA helicase activity	3678	75	9	9.22.E-07
helicase activity	4386	216	13	1.05.E-05
structural constituent of muscle	8307	80	8	1.48.E-05
single-stranded DNA binding	3697	165	11	1.96.E-05
actin filament binding	51015	265	14	2.11.E-05
single-stranded DNA-dependent ATPase activity	43142	14	4	3.20.E-05
DNA-dependent ATPase activity	8094	119	9	4.18.E-05
**Down**				

Biological Process*				
positive regulation of release of cytochrome c from mitochondria	90200	47	8	1.14E-08
apoptotic signaling pathway	97190	866	27	4.26E-08
cell aggregation	98743	28	6	1.93E-07
regulation of release of cytochrome c from mitochondria	90199	74	8	4.42E-07
regulation of apoptotic signaling pathway	2001233	556	19	1.26E-06
release of cytochrome c from mitochondria	1836	89	8	1.84E-06
positive regulation of apoptotic signaling pathway	2001235	269	13	1.58E-06
cartilage condensation	1502	26	5	3.74E-06
circulatory system process	3013	752	21	8.17E-06
blood circulation	8015	748	21	7.53E-06
Cellular component				
integral component of plasma membrane	5887	2245	40	5.25.E-05
extracellular space	5615	5099	73	5.53.E-05
intrinsic component of plasma membrane	31226	2332	41	5.63.E-05
extracellular region	5576	6459	87	7.42.E-05
extracellular region part	44421	5438	76	8.22.E-05
integrin alphaIIb-beta3 complex	70442	2	2	8.65.E-05
ciliary neurotrophic factor receptor complex	70110	10	3	9.15.E-05
plasma membrane part	44459	3838	58	1.01.E-04
Molecular Function				
spermidine binding	19809	5	3	7.86.E-06
diamine N-acetyltransferase activity	4145	6	3	1.56.E-05

* Omitted from the top 10
